# Heparinoid Cream Restores Epidermal Barrier Homeostasis and Attenuates Type 2 Inflammation in MC903-Induced Dermatitis

**DOI:** 10.3390/ijms27209360

**Published:** 2026-10-21

**Authors:** Ayaka Yasuda, Natsuko Saito-Sasaki, Reiko Hara, Yu Sawada

**Affiliations:** Department of Dermatology, University of Occupational and Environmental Health, 1-1 Iseigaoka, Yahatanishi-ku, Kitakyushu 807-8555, Fukuoka, Japan

**Keywords:** heparinoid cream, atopic dermatitis, inflammation, skin barrier

## Abstract

Epidermal barrier dysfunction and cutaneous inflammation are closely interconnected in inflammatory skin diseases. However, the molecular effects of heparinoid cream on inflamed skin remain incompletely understood. In this study, we investigated the effects of heparinoid cream on epidermal barrier homeostasis and cutaneous inflammation using an MC903-induced dermatitis model. Eight-week-old female BALB/c mice were treated topically with MC903 for 7 consecutive days, with or without concomitant application of heparinoid cream. Skin barrier function, histological changes, inflammatory markers, and gene expression were evaluated by transepidermal water loss measurements, histology, immunohistochemistry, quantitative PCR, and RNA sequencing. Therapeutic effects were also assessed by applying heparinoid cream after dermatitis had been established. RNA sequencing identified 816 differentially expressed genes following heparinoid cream treatment, including 146 upregulated and 670 downregulated genes. Upregulated genes were enriched in epidermal differentiation- and barrier-related pathways, whereas downregulated genes were enriched in immune and inflammatory pathways. Heparinoid cream significantly reduced transepidermal water loss and epidermal thickness, suppressed *Il13* and *Tslp* expression, and reduced epidermal KLK5 and TSLP expression. In parallel, expression of multiple epidermal differentiation- and barrier-associated genes, including members of the *Lce* family, *Flg*, and *Flg2*, was restored. Therapeutic application of heparinoid cream after establishment of dermatitis was also associated with improved barrier function, increased Flg expression, reduced Tslp mRNA expression, and decreased epidermal KLK5 and TSLP immunohistochemical staining. Secondary reanalysis of a previously published human keratinocyte RNA-seq dataset revealed changes in selected barrier- and innate immunity-associated genes following MPS treatment under non-inflammatory monolayer culture conditions. These findings demonstrate that heparinoid cream treatment is associated with concurrent improvements in epidermal barrier-related parameters and attenuation of type 2 inflammatory responses in MC903-induced dermatitis. These findings provide molecular insights into the changes associated with topical application of a heparinoid-containing formulation in inflamed skin, although the specific contribution of MPS remains to be established.

## 1. Introduction

The epidermal barrier is essential for maintaining cutaneous homeostasis by preventing excessive water loss and protecting the body from environmental insults [1]. Disruption of this barrier is a characteristic feature of inflammatory skin diseases and is closely linked to cutaneous immune activation [2,3]. Impaired epidermal differentiation and altered expression of structural proteins compromise barrier integrity, while inflammatory cytokines can further disrupt epidermal differentiation and barrier function [4]. Conversely, barrier disruption can enhance the production of epithelial-derived inflammatory mediators, thereby promoting cutaneous inflammation. Thus, epidermal barrier dysfunction and inflammation can mutually reinforce each other, forming a self-amplifying cycle that contributes to the development and persistence of inflammatory skin lesions [5].

Moisturizers are widely used to improve epidermal barrier dysfunction and constitute an important component of the management of dry and inflammatory skin conditions [6]. By increasing water content in the stratum corneum and reducing transepidermal water loss (TEWL), moisturizers can improve skin hydration and barrier function [7]. However, moisturizers comprise diverse formulations and active ingredients, and their biological effects on inflamed skin may not necessarily be explained solely by their physicochemical moisturizing properties [8]. Understanding whether commonly used moisturizers influence epidermal differentiation and inflammatory pathways at the molecular level may therefore provide additional insight into their therapeutic effects.

Heparinoid cream is a widely used topical moisturizer with water-retaining properties and is frequently applied to dry and barrier-impaired skin [9]. Although heparinoid-containing preparations have been shown to improve epidermal barrier function [9], mucopolysaccharide polysulfate (MPS) has also been reported to modulate tight junction-associated molecules in keratinocytes [10]. Notably, MPS has been reported to partially restore epidermal function and attenuate inflammation in filaggrin-deficient mice with calcipotriol-induced dermatitis [11]. However, the broader molecular responses elicited by heparinoid cream in inflamed skin remain incompletely understood, particularly with regard to its effects on epidermal differentiation and inflammatory pathways. Comprehensive analysis of these molecular effects may help clarify the biological basis underlying the therapeutic actions of heparinoid cream in inflammatory skin conditions.

Although previous studies have demonstrated that heparinoid-containing formulations improve epidermal barrier function and modulate barrier-associated gene expression, their effects on inflammatory signaling in the context of established skin inflammation remain incompletely characterized. In particular, the coordinated changes in epidermal differentiation, KLK5 and TSLP expression, and type 2 inflammatory responses following topical heparinoid treatment have not been fully elucidated.

In the present study, we investigated the effects of heparinoid cream on epidermal barrier homeostasis and cutaneous inflammation using an MC903-induced dermatitis model. We first performed unbiased transcriptomic analysis to characterize the molecular changes associated with heparinoid cream treatment and subsequently examined epidermal barrier-related and inflammatory pathways. We further evaluated whether heparinoid cream could improve barrier dysfunction and inflammatory changes when administered after dermatitis had already been established. Through these complementary approaches, we sought to define the molecular and functional effects of heparinoid cream in inflamed skin.

## 2. Results

### 2.1. Heparinoid Cream Markedly Alters the Transcriptomic Profile of MC903-Induced Dermatitis

Heparinoid cream is widely used as a moisturizer for the management of dry and barrier-impaired skin. However, the molecular effects of heparinoid cream on inflamed skin remain largely unexplored, and it is unclear whether its effects extend beyond improvement of skin hydration. To obtain an unbiased overview of the molecular changes induced by heparinoid cream under inflammatory conditions, we performed transcriptomic analysis of skin from mice with MC903-induced dermatitis treated with or without heparinoid cream.

**Figure 1:**
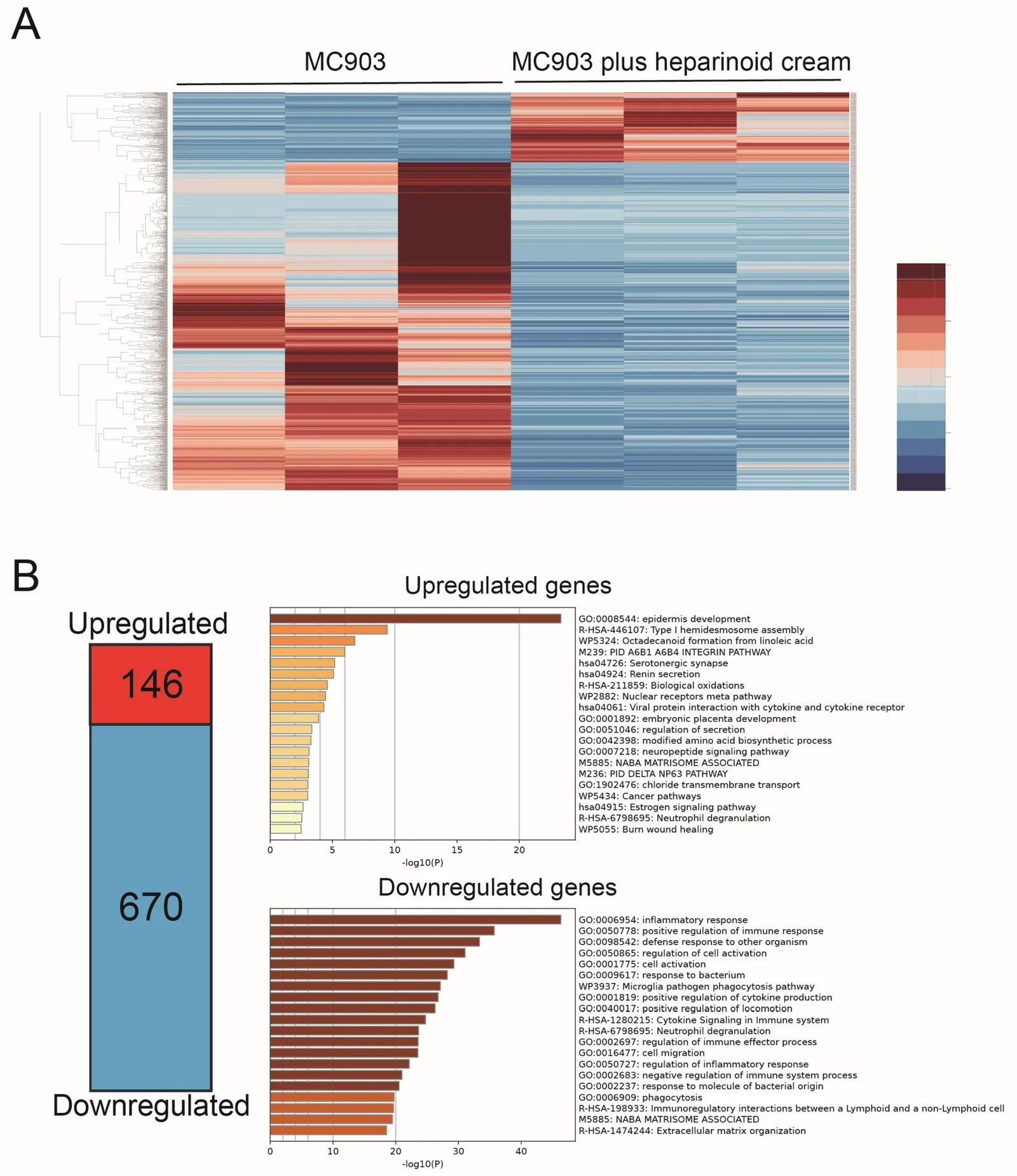
Transcriptomic changes induced by heparinoid cream in MC903-induced dermatitis. (**A**) Unsupervised hierarchical clustering of differentially expressed genes in skin samples from mice treated with MC903 alone or MC903 plus heparinoid cream. Each column represents an individual sample. (**B**) Differential gene expression and functional enrichment analyses comparing the MC903 and MC903 plus heparinoid cream groups. A total of 816 differentially expressed genes were identified using an adjusted *p* value < 0.05 and |log2FoldChange| ≥ 1, including 146 upregulated and 670 downregulated genes following heparinoid cream treatment. Upregulated genes were predominantly associated with epidermal differentiation and barrier-related processes, whereas downregulated genes were enriched in immune and inflammatory pathways. RNA sequencing was performed using three independent biological samples per group (n = 3 per group).

Unsupervised hierarchical clustering revealed clearly distinct gene expression profiles between the MC903 and MC903 plus heparinoid cream groups (Figure 1A). Differential gene expression analysis using an adjusted *p* value < 0.05 identified 816 differentially expressed genes, including 146 upregulated and 670 downregulated genes following heparinoid cream treatment (Figure 1B). These findings demonstrate substantial transcriptional changes associated with heparinoid cream application in inflamed skin.

We next performed functional enrichment analysis to determine the biological processes associated with these transcriptional changes. Notably, genes upregulated by heparinoid cream were enriched in pathways related to epidermal differentiation and barrier organization, including cornified envelope formation, establishment of the skin barrier, and type I hemidesmosome assembly (Figure 1B). Conversely, downregulated genes were strongly associated with immune and inflammatory processes, including positive regulation of immune response, inflammatory response, regulation of cell activation, and leukocyte migration (Figure 1B).

Collectively, these transcriptomic analyses identified two distinct biological signatures associated with heparinoid cream treatment: increased expression of genes related to epidermal differentiation and barrier formation, and decreased expression of genes associated with immune and inflammatory responses. These findings led us to further investigate how heparinoid cream affects cutaneous inflammation and epidermal barrier homeostasis in MC903-induced dermatitis.

### 2.2. Heparinoid Cream Attenuates MC903-Induced Skin Inflammation and Type 2 Inflammatory Responses

**Figure 2:**
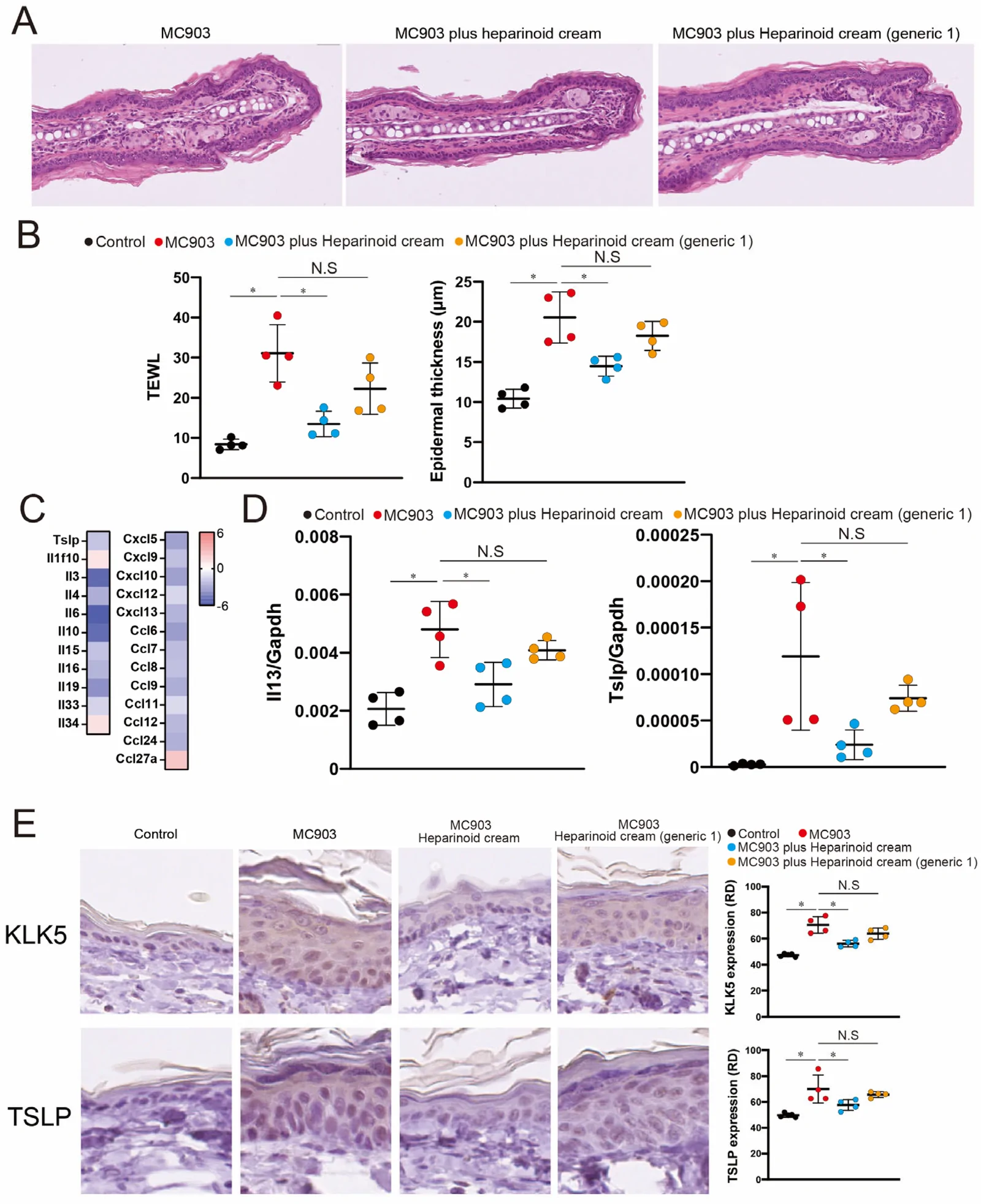
Heparinoid cream attenuates MC903-induced skin inflammation and type 2 inflammatory responses. (**A**) Representative hematoxylin and eosin (H&E)-stained sections of skin from the indicated treatment groups. (**B**) Transepidermal water loss (TEWL) and epidermal thickness in the indicated groups. (**C**) Heatmap showing the expression profiles of inflammation-associated genes identified by transcriptomic analysis in the MC903 and MC903 plus heparinoid cream groups. (**D**) Quantitative real-time PCR analysis of *Il13* and *Tslp* expression in skin from the indicated groups. (**E**) Representative immunohistochemical staining for KLK5 and TSLP in skin from the indicated treatment groups. Data are presented as mean ± SD. Statistical significance was assessed using one-way ANOVA followed by Tukey’s multiple-comparisons test. Comparisons between the control and MC903 groups and between the MC903 and heparinoid cream-treated groups are indicated in the figure. *p* < 0.05 was considered statistically significant. For Figure 2B,D,E, n = 4 mice per group. Each dot represents an individual mouse. * *p* < 0.05; N.S., not significant.

Transcriptomic analysis revealed that heparinoid cream downregulated multiple immune- and inflammation-related pathways in MC903-induced dermatitis. We therefore examined whether these transcriptional changes were accompanied by improvement in the inflammatory phenotype of the skin. To determine whether the observed effects were specific to the original formulation or were also reproduced by another heparinoid-containing product, we included a generic heparinoid cream as a comparator. Histological examination showed marked epidermal hyperplasia in MC903-treated skin, which was attenuated by concomitant treatment with heparinoid cream (Figure 2A). Consistent with these histological findings, MC903-induced increases in TEWL and epidermal thickness were significantly reduced by heparinoid cream treatment (Figure 2B).

To further characterize the inflammatory pathways affected by heparinoid cream, we analyzed the expression of inflammation-associated genes identified in the transcriptomic dataset. Heatmap analysis demonstrated distinct expression patterns of multiple cytokine- and chemokine-related genes between the MC903 and MC903 plus heparinoid cream groups (Figure 2C). Among these inflammatory pathways, we focused on type 2 inflammation, a major component of MC903-induced dermatitis. Quantitative PCR analysis demonstrated that Il13 and Tslp expression levels were significantly reduced in the original heparinoid cream-treated group compared with the MC903 group, whereas no statistically significant differences were observed in the generic formulation-treated group (Figure 2D).

We next examined epidermal KLK5 and TSLP, which are involved in the initiation and amplification of cutaneous inflammatory responses. Immunohistochemical analysis showed increased epidermal expression of KLK5 and TSLP following MC903 treatment, whereas their expression was markedly reduced by concomitant heparinoid cream treatment (Figure 2E).

Collectively, these findings demonstrate that heparinoid cream treatment was associated with improvement in the inflammatory phenotype of MC903-induced dermatitis, accompanied by reduced epidermal KLK5 and TSLP expression and attenuation of type 2 inflammatory responses.

### 2.3. Heparinoid Cream Restores Epidermal Differentiation and Barrier-Related Gene Expression

**Figure 3:**
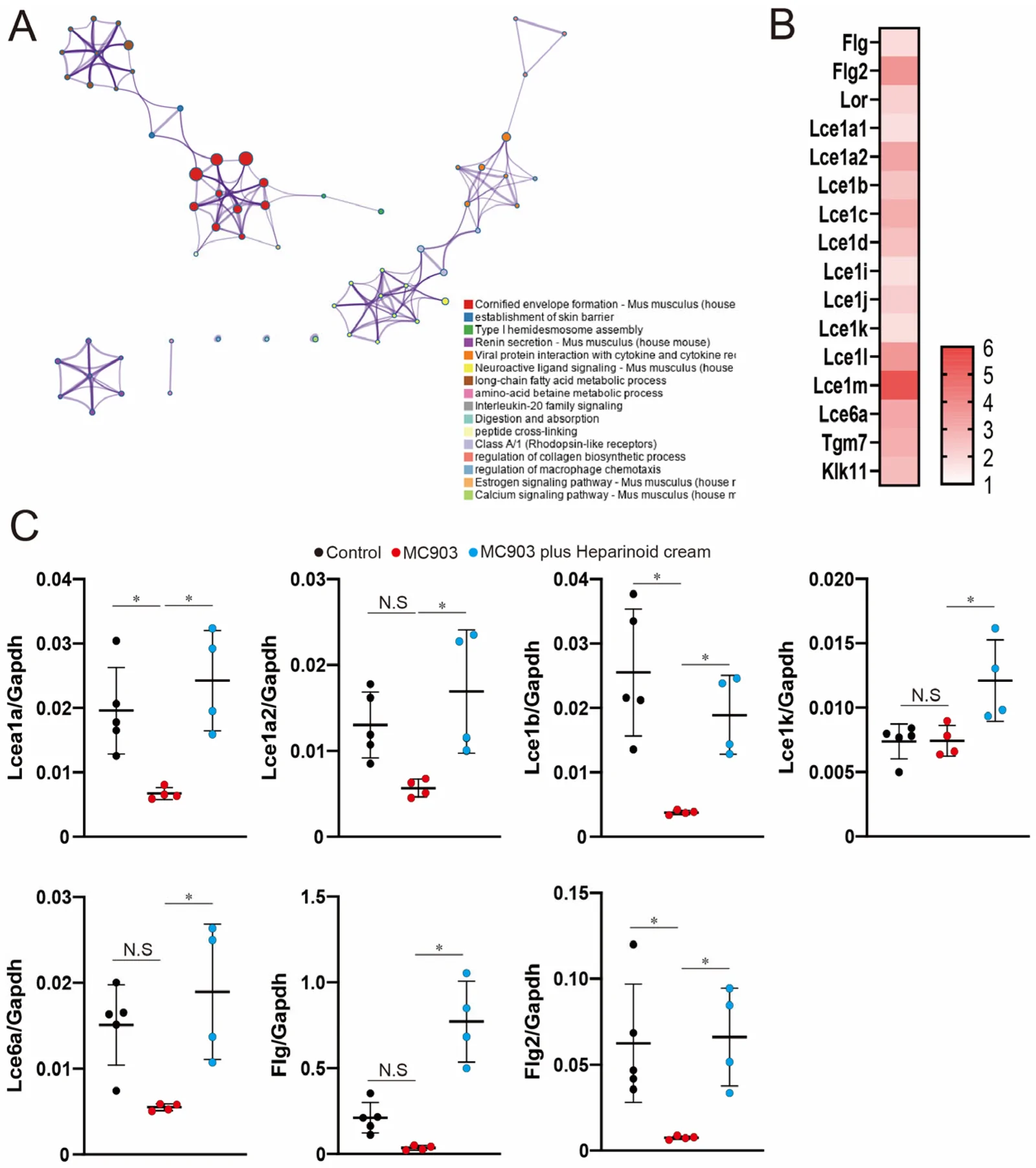
Heparinoid cream restores epidermal differentiation- and barrier-related gene expression in MC903-induced dermatitis. (**A**) Network analysis of biological processes enriched among differentially expressed genes following heparinoid cream treatment. Functionally related pathways associated with epidermal differentiation and barrier formation, including cornified envelope formation and establishment of the skin barrier, are shown. (**B**) Heatmap showing fold changes in the expression of epidermal differentiation- and barrier-associated genes in the MC903 plus heparinoid cream group relative to the MC903 group, which was set to 1. (**C**) Quantitative real-time PCR analysis of *Lce1a1*, *Lce1a2*, *Lce1b*, *Lce1k*, *Lce6a*, *Flg*, and *Flg2* expression in skin from the indicated groups. Data are presented as mean ± SD. Statistical significance was assessed using one-way ANOVA followed by Tukey’s multiple-comparisons test. Comparisons between the control and MC903 groups and between the MC903 and heparinoid cream-treated groups are indicated in the figure. *p* < 0.05 was considered statistically significant. For Figure 3C, n = 4–5 mice per group, as indicated by the individual data points. * *p* < 0.05; N.S, not significant.

In addition to the suppression of inflammatory pathways, transcriptomic analysis revealed prominent enrichment of epidermal differentiation- and barrier-related pathways among genes upregulated by heparinoid cream treatment. To further characterize these transcriptional changes, we performed pathway network analysis of the differentially expressed genes. This analysis identified closely interconnected functional clusters associated with epidermal differentiation and barrier formation, including cornified envelope formation and establishment of the skin barrier (Figure 3A).

We therefore focused on genes involved in the formation and maintenance of the epidermal barrier. Transcriptomic analysis revealed coordinated changes in multiple barrier-associated genes, particularly members of the late cornified envelope (Lce) family, as well as filaggrin (Flg) and filaggrin 2 (Flg2) (Figure 3B). Notably, these genes showed reduced expression in MC903-induced dermatitis and increased expression following heparinoid cream treatment.

To validate these transcriptomic findings, we examined the expression of representative epidermal barrier-related genes by quantitative PCR. Quantitative PCR analysis revealed that the expression levels of several epidermal barrier-associated genes were reduced following MC903 treatment, with statistically significant reductions observed for Lce1a1, Lce1b, and Flg2 compared with the control group. Notably, heparinoid cream treatment was associated with significantly increased expression of all seven examined barrier-associated genes compared with the MC903 group (Figure 3C).

Together, these results demonstrate that heparinoid cream treatment is associated with increased expression of multiple epidermal differentiation- and barrier-associated genes in MC903-induced dermatitis, consistent with improvement in epidermal barrier-related transcriptional profiles.

### 2.4. Changes in Epidermal Barrier Function and Inflammatory Markers Following Heparinoid Cream Application After MC903 Withdrawal

**Figure 4:**
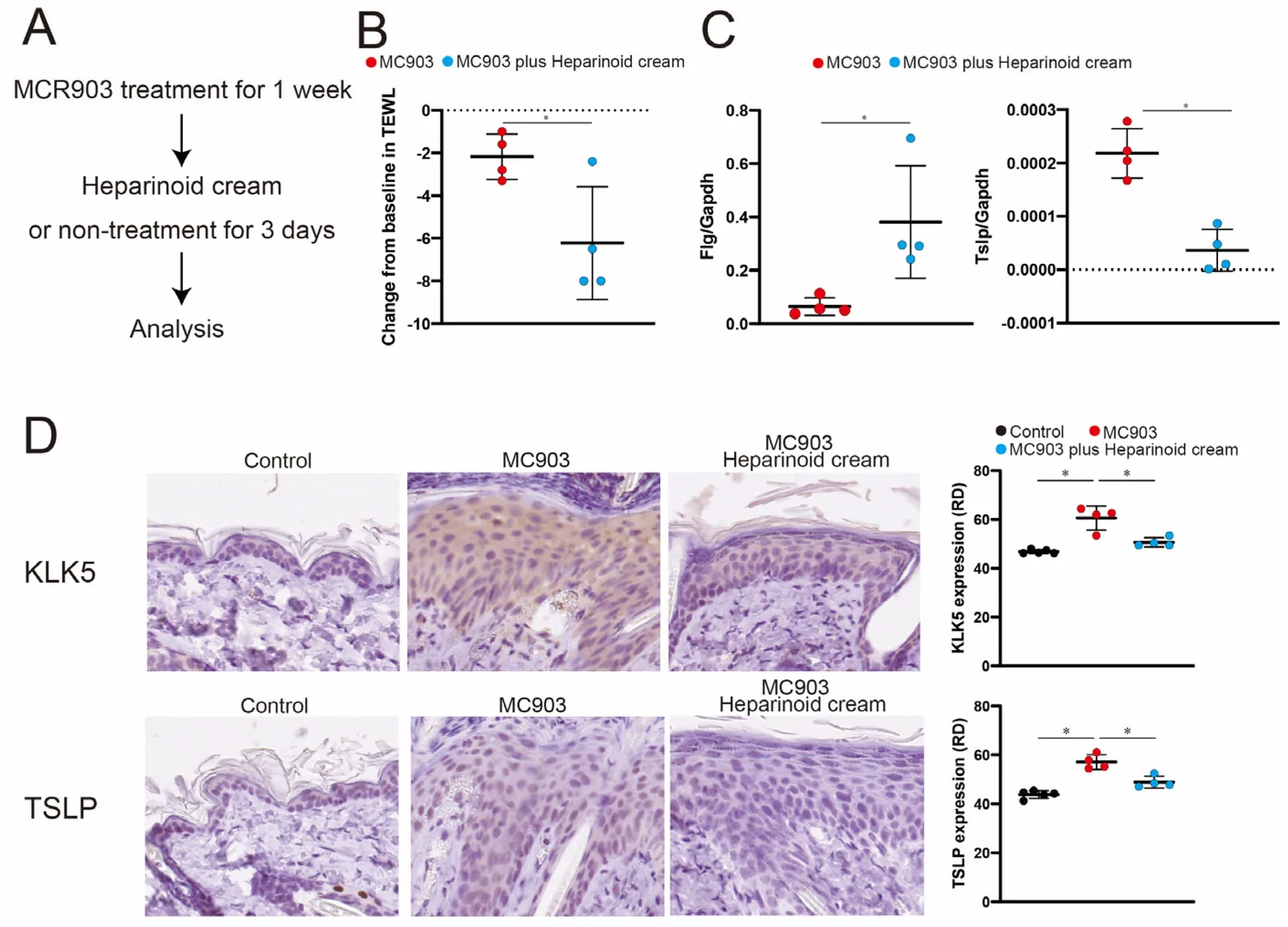
Changes in epidermal barrier function and inflammatory markers following heparinoid cream application after MC903 withdrawal. (**A**) Schematic representation of the experimental protocol. Dermatitis was induced by topical application of MC903 for 7 consecutive days. After discontinuation of MC903, heparinoid cream was applied for an additional 3 days. (**B**) Changes in transepidermal water loss (TEWL) from day 7 to day 10 following MC903 discontinuation. TEWL changes were compared between the untreated withdrawal control group and the heparinoid cream-treated group. (**C**) Quantitative real-time PCR analysis of Flg and Tslp expression in skin from the indicated groups. (**D**) Representative immunohistochemical staining and quantification of epidermal KLK5 and TSLP in untreated healthy control mice, untreated MC903 withdrawal control mice, and heparinoid cream-treated mice following MC903 withdrawal. Data are presented as mean ± SD. Statistical significance was assessed using Welch’s unpaired *t*-test for two-group comparisons (**B**,**C**) and ordinary one-way ANOVA followed by Tukey’s multiple-comparisons test for three-group comparisons (**D**). For Figure 4B–D, n = 4 mice per group. Each dot represents an individual mouse. * *p* < 0.05.

Our findings thus far demonstrated that concomitant application of heparinoid cream attenuated MC903-induced inflammation and preserved epidermal barrier-related gene expression. We therefore examined changes in epidermal barrier function and inflammatory markers following heparinoid cream application after the establishment of dermatitis. Mice were treated with MC903 for 7 days, followed by discontinuation of MC903 and topical application of heparinoid cream for an additional 3 days (Figure 4A).

Although TEWL decreased following MC903 withdrawal in both groups, the reduction from day 7 to day 10 was significantly greater in the heparinoid cream-treated group than in the untreated withdrawal control group (Figure 4B). Furthermore, quantitative real-time PCR analysis demonstrated significantly higher Flg expression and significantly lower Tslp expression in the heparinoid cream-treated group compared with the untreated withdrawal control group (Figure 4C). Immunohistochemical analysis also revealed lower epidermal KLK5 and TSLP expression in the heparinoid cream-treated group compared with the untreated withdrawal control group (Figure 4D). These findings suggest that heparinoid cream application was associated with additional improvements in epidermal barrier function and inflammatory markers beyond spontaneous recovery following MC903 withdrawal.

Collectively, these findings indicate that topical application of heparinoid cream following the establishment of MC903-induced dermatitis was associated with reduced TEWL, increased Flg expression, decreased Tslp mRNA expression, and reduced epidermal KLK5 and TSLP immunohistochemical staining. Importantly, comparison with the untreated withdrawal control group demonstrated significantly greater improvement in TEWL, higher Flg expression, and lower Tslp expression following heparinoid cream treatment, supporting an additional beneficial effect beyond spontaneous recovery after MC903 discontinuation.

### 2.5. Secondary Reanalysis of Published Human Keratinocyte RNA-Seq Data Reveals Changes in Barrier- and Innate Immunity-Associated Gene Expression

**Figure 5:**
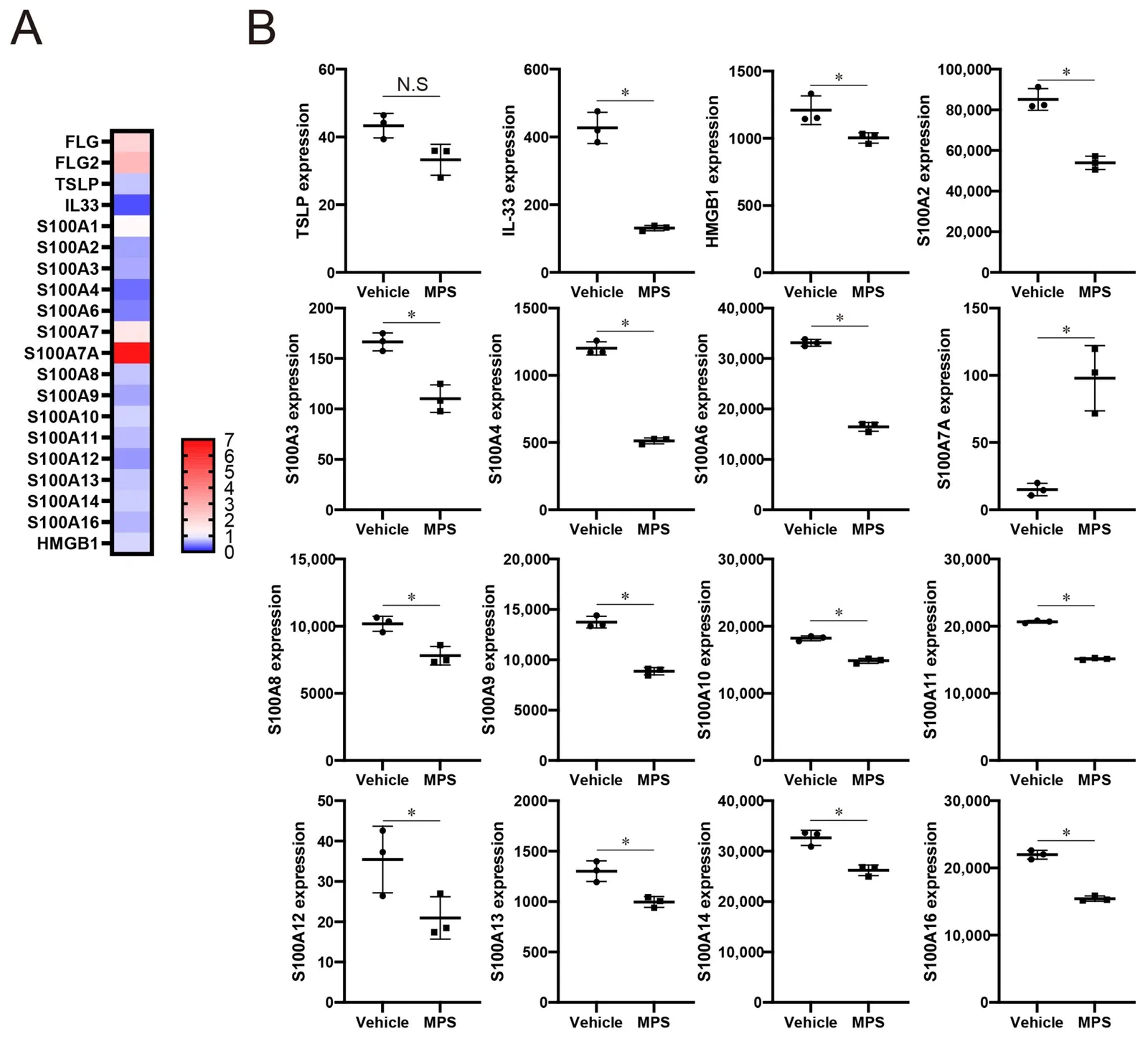
Exploratory reanalysis of barrier- and innate immunity-associated gene expression in human epidermal keratinocytes following MPS treatment. (**A**) Heatmap showing fold changes in selected epidermal barrier- and innate immunity-associated genes in monolayer-cultured human epidermal keratinocytes following treatment with mucopolysaccharide polysulfate (MPS, 1 μg/mL) for 24 h. Fold changes were calculated from DESeq2-normalized counts relative to the vehicle-treated control group, which was set to 1. (**B**) Expression levels of selected barrier-, inflammation-, and innate immunity-associated genes in monolayer-cultured human epidermal keratinocytes treated with vehicle or MPS (1 μg/mL) for 24 h. Gene expression levels are presented as DESeq2-normalized counts. Data are shown as mean ± standard deviation (SD), with three independent biological samples per group (n = 3). Each dot represents an independent biological sample. Differential expression analysis was performed using DESeq2 based on raw count data, and *p* values were adjusted for multiple testing using the Benjamini–Hochberg method. * Adjusted *p* < 0.05; N.S., not significant. All data were obtained through secondary reanalysis of a previously published RNA-seq dataset (GSE268656).

To explore the transcriptional responses associated with MPS treatment in human keratinocytes, we performed a secondary reanalysis of a previously published RNA-seq dataset (GSE268656). All analyses presented in Figure 5 were based on monolayer-cultured human epidermal keratinocytes. Exploratory analysis revealed changes in the expression of selected epidermal barrier- and innate immunity-associated genes following MPS treatment, including FLG, FLG2, IL33, and members of the S100 family (Figure 5A). Individual gene expression analyses further revealed changes in IL33, several S100 family members, and HMGB1, whereas the reduction in TSLP expression did not reach statistical significance (Figure 5B). These observations provide complementary information regarding keratinocyte-associated transcriptional responses to MPS. However, they were derived from secondary analysis of previously published data and do not constitute direct validation of the findings obtained in the mouse dermatitis model.

## 3. Discussion

In this study, we investigated the effects of heparinoid cream on epidermal barrier homeostasis and cutaneous inflammation using an MC903-induced dermatitis model. Unbiased transcriptomic analysis revealed broad changes in the cutaneous transcriptome following heparinoid cream treatment, characterized by upregulation of epidermal differentiation- and barrier-related pathways and downregulation of immune and inflammatory pathways. These transcriptomic changes were accompanied by improved barrier function, attenuation of histological inflammation, reduced type 2 inflammatory responses, reduced epidermal KLK5 and TSLP expression, and restoration of multiple epidermal barrier-associated genes, including members of the *Lce* family, *Flg*, and *Flg2*. Importantly, beneficial effects were also observed when heparinoid cream was applied after dermatitis had already been established. Collectively, these findings indicate that treatment with the heparinoid-containing formulation was associated with concurrent improvements in epidermal barrier-related parameters and inflammatory markers, although the relative contributions of MPS and other formulation components remain to be determined [12].

Heparinoid-containing topical formulations are widely used to improve dry skin and impaired skin barrier function. Their clinical benefits have traditionally been attributed primarily to their moisturizing properties, including enhancement of stratum corneum hydration. Previous experimental studies, however, have suggested that heparinoid-containing formulations may also influence epidermal barrier homeostasis at the molecular level [12,13]. Topical application of a heparinoid-containing cream has been shown to accelerate epidermal permeability barrier recovery and increase stratum corneum hydration, accompanied by altered expression of genes involved in epidermal differentiation and lipid synthesis [12]. In addition, heparinoid-containing cream has been reported to attenuate glucocorticoid-induced epidermal barrier dysfunction together with changes in the expression of barrier-related genes [14]. Nevertheless, the molecular effects of heparinoid-containing formulations in the context of active cutaneous inflammation remain incompletely understood. In the present study, transcriptomic analysis revealed two complementary responses to heparinoid cream: enhancement of epidermal differentiation- and barrier-related pathways and attenuation of immune and inflammatory pathways. This coordinated response suggests that the biological effects associated with heparinoid treatment in inflamed skin may extend beyond improvement in skin hydration alone.

One of the most prominent effects of heparinoid cream was the restoration of epidermal differentiation-associated genes. MC903 markedly reduced the expression of several members of the *Lce* family as well as *Flg* and *Flg2*, whereas heparinoid treatment restored their expression. Filaggrin is a major structural component of the epidermal barrier and plays an essential role in terminal keratinocyte differentiation and maintenance of stratum corneum homeostasis [15,16]. Late cornified envelope proteins are also key constituents of the cornified envelope and contribute to epidermal barrier formation [17]. Therefore, the coordinated restoration of these genes suggests that heparinoid cream may promote recovery of a broader epidermal differentiation program rather than simply modulating individual barrier molecules. This interpretation is further supported by pathway analyses demonstrating enrichment of cornified envelope formation and establishment of the skin barrier among genes upregulated following heparinoid treatment.

Our findings also indicate a close relationship between improvement in epidermal barrier homeostasis and attenuation of cutaneous inflammation. Epidermal barrier disruption can promote inflammatory responses through the release of epithelial-derived mediators, while inflammation itself can further impair epidermal differentiation and barrier function, thereby establishing a self-amplifying barrier–inflammation cycle [18]. In the present study, heparinoid cream reduced TEWL and epidermal hyperplasia, accompanied by decreased expression of inflammation-associated transcripts. In particular, *Il13* and *Tslp* expression was reduced following heparinoid treatment. IL-13 is a central type 2 cytokine that can impair epidermal differentiation and barrier function [19], whereas TSLP produced by epidermal keratinocytes promotes type 2 immune responses [20]. These findings demonstrate concurrent improvement in epidermal barrier-associated parameters and reduction in type 2 inflammatory markers following heparinoid cream treatment. However, the present study does not establish whether improvement in barrier function leads to attenuation of inflammation or whether reduced inflammation facilitates barrier recovery.

The reduction in epidermal KLK5 expression provides an additional potential link between barrier regulation and inflammatory responses. KLK5 is a serine protease expressed in the epidermis and has important roles in stratum corneum homeostasis and desquamation [21]. Excessive epidermal protease activity can disrupt barrier integrity and promote inflammatory signaling, including pathways leading to TSLP production [21,22,23]. In our model, MC903 increased epidermal KLK5 and TSLP expression, whereas both were reduced by heparinoid cream. The concurrent reductions in KLK5 and TSLP expression suggest a possible association between epidermal protease-related responses and inflammatory changes following heparinoid cream treatment. However, these observations do not demonstrate direct regulation of TSLP by KLK5 or establish the functional involvement of the KLK5–TSLP pathway in the observed inflammatory changes. Further studies involving pharmacological inhibition or genetic manipulation of KLK5 and TSLP are required to clarify their causal contributions.

Previous studies have demonstrated increased epidermal KLK5 protein expression in atopic dermatitis lesions [23]. In contrast, a previous study showed that KLK7 mRNA, but not KLK5 mRNA, was significantly upregulated in both human atopic dermatitis lesions and MC903-induced murine dermatitis [24]. Furthermore, Morizane et al. demonstrated that KLK5 mRNA expression and secreted protein accumulation exhibited different temporal patterns following vitamin D3 stimulation of keratinocytes [25]. These observations suggest that KLK5 mRNA levels at a single time point may not fully reflect protein abundance, supporting the complementary value of assessing epidermal KLK5 protein expression.

Importantly, the effects of heparinoid cream were not restricted to concomitant application during dermatitis induction. When treatment was initiated after MC903-induced dermatitis had already developed, heparinoid cream treatment was associated with reduced TEWL, increased Flg expression, decreased Tslp mRNA expression, and reduced epidermal KLK5 and TSLP immunohistochemical staining. These observations indicate that changes in barrier-related and inflammatory parameters were also detected during the recovery period following MC903 discontinuation. Although spontaneous recovery occurred after MC903 withdrawal, the inclusion of an untreated withdrawal control group allowed us to compare the additional changes associated with heparinoid cream application. Nevertheless, the absence of a vehicle-matched control prevents us from distinguishing formulation-specific effects from those attributable to MPS itself. Although moisturizers and emollients are widely used in the management of inflammatory skin diseases [26,27], the experimental treatment conditions used in the present mouse model differ from those typically employed in clinical practice. Therefore, the observed molecular and barrier-related changes should not be directly extrapolated to clinical therapeutic efficacy.

The downregulation of immune-related transcripts observed in whole-skin RNA sequencing should be interpreted with caution. Because skin samples contain heterogeneous cell populations, these transcriptional changes may partly reflect a reduction in inflammatory cell infiltration following improvement in dermatitis rather than direct suppression of inflammatory signaling pathways within individual cells. In addition, restoration of epidermal barrier homeostasis may secondarily attenuate cutaneous inflammation. Therefore, the present findings do not establish whether the observed reduction in immune-related gene expression results from direct modulation of inflammatory pathways, changes in immune cell composition, or secondary effects of barrier restoration. Further cell-type-specific and functional analyses are required to clarify these mechanisms. Furthermore, the present findings do not establish the safety or efficacy of the experimental application conditions in humans and should not be interpreted as supporting changes in the recommended clinical dosage, application frequency, or indications of heparinoid-containing formulations. Clinical use should remain consistent with established prescribing information.

Previous studies have suggested that the effects of topical heparinoid preparations may be influenced by formulation-specific properties. In particular, a direct comparison of original and generic heparinoid creams demonstrated differences in film-forming ability among formulations [28]. These findings provided a rationale for including a generic heparinoid formulation as a comparator in the present study. However, because direct statistical comparisons between the original and generic formulations were not performed in the present study, no conclusions regarding their relative efficacy can be drawn. Further studies incorporating direct comparative analyses are needed to clarify whether formulation-specific properties contribute to differences in biological effects. Importantly, because a matched vehicle formulation lacking MPS was not available, the present study cannot distinguish the effects of MPS itself from those of the cream base or other formulation components. Therefore, the observed biological changes should be interpreted as effects associated with the complete heparinoid-containing formulation rather than the isolated pharmacological activity of MPS.

Several limitations should be acknowledged. First, the MC903-induced dermatitis model does not fully reproduce the complexity of human inflammatory skin diseases, and the effects observed in mice require validation in human skin [29]. Furthermore, the amount and application conditions of the topical formulation used in the present mouse experiments differ from those typically employed in clinical practice. Differences in skin structure, relative surface area, formulation exposure, and inflammatory conditions may influence treatment responses. Therefore, these experimental findings should be interpreted as preclinical observations rather than direct evidence of clinical efficacy. In addition, the human keratinocyte findings were derived from secondary reanalysis of a previously published RNA-seq dataset rather than newly conducted experiments. Importantly, these data were obtained from monolayer-cultured keratinocytes treated with MPS in the absence of experimentally induced inflammation. Although changes in selected epidermal barrier- and innate immunity-associated genes were observed, these findings do not establish direct anti-inflammatory effects under inflammatory conditions. Furthermore, monolayer cultures do not reproduce the complex cellular and inflammatory microenvironment of human skin in vivo. Therefore, these observations should be regarded as complementary transcriptional findings rather than direct validation of the results obtained in the MC903-induced dermatitis model.

Second, although transcriptomic, molecular, and functional analyses demonstrated concurrent changes in epidermal barrier-associated parameters and inflammatory markers, the present study does not establish the temporal or causal relationships between these processes. Specifically, we cannot determine whether restoration of epidermal barrier function contributes to attenuation of inflammation, whether reduced inflammation facilitates barrier recovery, or whether these responses occur in parallel. Third, although reduced KLK5 and TSLP expression was observed following heparinoid cream treatment, these findings do not establish direct regulation of the KLK5–TSLP pathway or its functional contribution to the observed inflammatory changes. Further studies involving pharmacological inhibition or genetic manipulation of KLK5 and TSLP will be necessary to establish their mechanistic roles. Although Tslp mRNA expression was evaluated in the post-dermatitis treatment experiment and was significantly reduced following heparinoid cream treatment, corresponding Klk5 mRNA expression and KLK5 enzymatic activity were not assessed. Therefore, the present findings do not establish the functional involvement of the KLK5–TSLP pathway in the observed treatment-associated changes. Finally, the lack of a vehicle-matched control group represents an important limitation of this study. Because the corresponding cream base without MPS was unavailable, we could not determine whether the observed effects were attributable specifically to MPS or to other formulation components, including excipients and the physicochemical properties of the cream base. In addition, the relatively small sample size and variability in Tslp expression observed in Figure 2D may have limited the statistical power to detect differences between the MC903- and generic formulation-treated groups. Therefore, the absence of statistically significant differences in the generic formulation group should be interpreted cautiously. Future studies incorporating appropriate vehicle-matched controls are necessary to distinguish the specific contribution of MPS from formulation-dependent effects.

## 4. Materials and Methods

### 4.1. Animals

Eight-week-old female BALB/c mice were purchased from Japan SLC, Inc. (Shizuoka, Japan). Mice were maintained under specific pathogen-free conditions with free access to food and water. All animal experiments were performed in accordance with institutional guidelines for animal care and use and were approved by the Institutional Animal Care and Use Committee of the University of Occupational and Environmental Health, Japan (approval number: AE22-022, AE26-006).

### 4.2. MC903-Induced Dermatitis Model and Topical Treatment

MC903-induced dermatitis was induced as previously described with modifications [30]. Briefly, MC903 (calcipotriol; 2 nmol in 20 μL ethanol) was topically applied to the shaved dorsal skin once daily for 7 consecutive days. For concomitant treatment experiments, heparinoid cream (Hirudoid Soft Ointment 0.3%, Maruho Co., Ltd., Osaka, Japan) (30 mg) was topically applied to the MC903-treated dorsal skin during the 7-day induction period. A generic heparinoid cream was used as a comparator where indicated. On day 7, skin samples were collected for histological examination, immunohistochemistry, RNA sequencing, and quantitative real-time PCR analyses.

### 4.3. Therapeutic Treatment of Established Dermatitis

To evaluate the therapeutic effects of heparinoid cream after the establishment of dermatitis, mice were initially treated with MC903 for 7 consecutive days. Following discontinuation of MC903, mice were divided into two groups. One group received no further topical treatment for 3 days and served as an untreated withdrawal control, whereas the other group received topical heparinoid cream once daily for the same period. Both groups were evaluated on day 10. TEWL was measured on days 7 and 10, and skin samples were collected on day 10 for gene expression and immunohistochemical analyses. For immunohistochemical analysis of KLK5 and TSLP, an additional untreated healthy control group was included, resulting in three groups for this analysis.

### 4.4. Measurement of Transepidermal Water Loss

Epidermal barrier function was evaluated by measuring TEWL using a VAPO SCAN AS-VT100 (Asahi Biomed, Tokyo, Japan). TEWL measurements were performed on the skin at the indicated time points during the experimental period. The probe was placed directly on the skin surface, and water loss from the epidermis was determined according to the manufacturer’s instructions. Care was taken to perform the measurements consistently at the same anatomical site across the experimental groups. TEWL values were recorded as g/m^2^/h and used as an indicator of epidermal barrier function, with higher TEWL values reflecting greater impairment of the skin barrier.

### 4.5. Histological Analysis

Skin samples were fixed in 10% neutral-buffered formalin, embedded in paraffin, sectioned, and stained with hematoxylin and eosin (H&E). Epidermal thickness was measured in five different fields per section at ×200 magnification, and the mean value obtained from the five fields was used as the representative value for each mouse.

### 4.6. Immunohistochemistry

Paraffin-embedded skin sections were deparaffinized and subjected to antigen retrieval in 0.01 M citrate buffer (pH 6.0) by autoclaving at 120 °C for 5 min. After blocking, sections were incubated with primary antibodies against KLK5 (Anti-Kallikrein-5 Antibody/KLK5, ABC470; Merck, Darmstadt, Germany) and TSLP (Anti-TSLP antibody, ab188766; Abcam, Cambridge, UK) for 2 h at room temperature. Immunostaining was performed using the EnVision+ Dual-link system (Dako Cytomation, Glostrup, Denmark), with 3,3′-diaminobenzidine (DAB) as the chromogen to visualize antibody binding. Sections were counterstained with hematoxylin.

The intensity of immunohistochemical staining was quantified using the red density (RD) method as previously described [31,32]. Digital images of the stained sections were exported as JPEG files, and three different areas of the epidermis were selected for each sample. The selected areas were analyzed using red-channel histograms. The histogram intensity was expressed on a scale of 0–255, ranging from black (0) to white (255). The mean red-channel intensity was determined for each area, and the values obtained from the three areas were averaged. RD was calculated as 255 minus the mean red-channel intensity (RD = 255 − mean red-channel intensity), with higher RD values representing greater immunohistochemical staining intensity.

### 4.7. RNA Extraction and RNA Sequencing

Total RNA was extracted from skin samples using the PureLink RNA Mini Kit (Thermo Fisher Scientific, Waltham, MA, USA) according to the manufacturer’s instructions. RNA sequencing and subsequent bioinformatic analyses were performed by Rhelixa, Inc. (Tokyo, Japan). RNA-seq libraries were prepared using the NEBNext Poly(A) mRNA Magnetic Isolation Module and NEBNext Ultra II Directional RNA Library Prep Kit (New England Biolabs) and sequenced on an Illumina NovaSeq platform using 150-bp paired-end sequencing. Raw sequencing reads were subjected to quality control using FastQC (v0.12.1) and trimmed using Trimmomatic. The processed reads were aligned to the mouse reference genome using HISAT2, and gene-level read counts were obtained using featureCounts. Differential expression analysis was performed using DESeq2. *p* values were adjusted for multiple testing using the Benjamini–Hochberg method. Genes with an absolute log2 fold change (|log2FoldChange|) ≥ 1 and an adjusted *p* value < 0.05 were considered differentially expressed.

### 4.8. Functional Enrichment and Pathway Network Analyses

Functional enrichment and pathway network analyses were performed using Metascape. Upregulated and downregulated differentially expressed genes were analyzed separately to identify significantly enriched biological processes. Enriched terms were grouped according to their functional similarity, and representative terms were visualized to characterize the biological processes associated with heparinoid cream treatment. Network analysis of enriched terms was performed using Metascape to identify functionally related clusters.

### 4.9. Quantitative Real-Time PCR

Total RNA was extracted from skin samples using the PureLink RNA Mini Kit (Thermo Fisher Scientific) according to the manufacturer’s instructions. Purified total RNA was reverse-transcribed into complementary DNA (cDNA) using the Verso cDNA Synthesis Kit (Thermo Fisher Scientific). Quantitative real-time PCR was performed using PowerUp SYBR Green Master Mix (Thermo Fisher Scientific) with the ABI Prism 7000 Sequence Detection System (Applied Biosystems). Expression levels of *Il13*, *Tslp*, *Lce1a1*, *Lce1a2*, *Lce1b*, *Lce1k*, *Lce6a*, *Flg*, and *Flg2* were normalized to *Gapdh*. Relative gene expression was calculated using the 2^−ΔΔCt method. Primer sequences are provided in Supplementary Table S1.

### 4.10. Reanalysis of Previously Published Human Keratinocyte Data

We performed a secondary reanalysis of a previously published RNA-seq dataset (GSE268656) from our earlier study [33]. The analyzed samples were derived from monolayer-cultured human epidermal keratinocytes treated with vehicle or mucopolysaccharide polysulfate (MPS, 1 μg/mL) for 24 h under conditions without experimentally induced inflammatory stimulation. Three independent biological samples were included in each group. Raw gene count data were obtained from the publicly available dataset and analyzed using DESeq2. Genes with at least 10 counts in three or more samples were retained for differential expression analysis. Gene counts were normalized using size factors estimated by DESeq2. Differential expression was assessed using a negative binomial model, and *p* values were adjusted for multiple testing using the Benjamini–Hochberg method. For visualization, normalized counts were used to generate individual gene expression plots. Fold changes for the heatmap were calculated as the ratio of mean normalized counts in the MPS-treated group to those in the vehicle-treated control group. An adjusted *p* value < 0.05 was considered statistically significant. No additional in vitro experiments were conducted as part of the present study.

### 4.11. Statistical Analysis

Statistical analyses were performed using GraphPad Prism version 9.5.0 (GraphPad Software, San Diego, CA, USA). Comparisons among multiple groups were performed using one-way analysis of variance (ANOVA) followed by Tukey’s multiple-comparisons test. Two-group comparisons were performed using Welch’s unpaired two-tailed *t*-test, without assuming equal variances. A two-sided *p* value < 0.05 was considered statistically significant. Statistical analysis of the publicly available human keratinocyte RNA-seq dataset was performed separately using DESeq2, as described in Section 4.10. All experimental data are presented as mean ± standard deviation (SD), unless otherwise indicated.

## 5. Conclusions

In conclusion, our study demonstrates that heparinoid cream is associated with coordinated restoration of epidermal barrier-related transcriptional programs and attenuation of cutaneous inflammatory responses in MC903-induced dermatitis. Heparinoid treatment restored multiple epidermal differentiation-associated genes, reduced KLK5 and TSLP expression and type 2 inflammatory responses, and improved barrier dysfunction even when administered after dermatitis had been established. These findings provide new insight into the molecular and functional changes associated with heparinoid cream treatment in inflamed skin, although the causal relationships between epidermal barrier improvement and attenuation of inflammation remain to be elucidated. Further clinical and translational studies are required to determine whether similar biological responses occur under clinically relevant application conditions in patients with inflammatory skin diseases.

## Data Availability

The RNA-sequencing data generated in this study have been deposited in the NCBI Sequence Read Archive (SRA) under BioProject accession number PRJNA1526448.

## Supplementary Materials

The following supporting information can be downloaded at: https://www.mdpi.com/article/10.3390/ijms27209360/s1.
