# Supplementary material for: Heparinoid Cream Restores Epidermal Barrier Homeostasis and Attenuates Type 2 Inflammation in MC903-Induced Dermatitis
Source: Int J Mol Sci. 2026 Oct 21;27(20):9360. doi: 10.3390/ijms27209360 (PMC13705079; doi:10.3390/ijms27209360)
Supplement: Supplementary file 1 [file ijms-27-09360-s001.zip › ijms-4609738-supplementary.pdf]

Supplementary Table S1. Primer sequences used for quantitative real-time PCR

Gene	Forward primer (5′ –3′)	Reverse primer (5′ –3′)
Il13	CCTGGCTCTTGCTTGCCTT	GGTCTTGTGTGATGTTGCTCA
Tslp	ACGGATGGGGCTAACTTACAA	AGTCCTCGATTTGCTCGAACT
Lce1a1	AGCCATGTTCAGTGGACCTT	AAACCCAGCAATAGGGGAACA
Lce1a2	CCATGTTCAGTGGACCCTCC	ACAGGTCCTTCAAAAGGGGAC
Lce1b	GCACTTCAGACAAGCTGTGG	GCAGAGGATCACCAAGAGTCC
Lce1k	GTTCAGAAGACTGCCCCTGC	ACACAGAAGGGGTAGCAACA
Lce6a	CCAAACCTCTCTTTGAGTACCTG	GCCTCAGCCTTTTTGAGTAGGAA
Flg	ATGTCCGCTCTCCTGGAAAG	TGGATTCTTCAAGACTGCCTGTA
Flg2	CTAGAGGGCATGAGTGTAGTCA	CAAGACTGGACAGTTGGCTGG
Gapdh	AGGTCGGTGTGAACGGATTTG	TGTAGACCATGTAGTTGAGGTCA
